# Patient involvement in the development of a psychosocial cancer rehabilitation intervention: evaluation of a shared working group with patients and researchers

**DOI:** 10.1186/s40900-018-0106-2

**Published:** 2018-08-06

**Authors:** Eva Rames Nissen, Vibeke Bregnballe, Mimi Yung Mehlsen, Anne Kathrine Østerby Muldbjerg, Maja O’Connor, Kirsten Elisabeth Lomborg

**Affiliations:** 1 Unit for Psychooncology and Health Psychology, Department of Psychology, Aarhus University, Bartholins Allé 11, Bld. 1350, DK-8000 Aarhus C, Denmark; 2 Research Programme for Patient Involvement, Department of Clinical Medicine, Aarhus University and Aarhus University Hospital, Nørrebrogade 44, Bld. 12A, 1, DK-8000 Aarhus C, Denmark; 3 Department of Clinical Medicine, Aarhus University, Incuba/Skejby, Bld. 2, Palle Juul-Jensens Boulevard 82, DK-8200 Aarhus N, Denmark

**Keywords:** PPI, Patient and public involvement in research, Internet-delivered interventions, Cancer survivorship, Cancer rehabilitation

## Abstract

**Plain English summary:**

The aim of this paper is to present our experiences from a shared working group (SWG) with patient representatives and researchers. The SWG collaborated on developing a psychosocial cancer rehabilitation intervention for women treated for breast cancer and men treated for prostate cancer and on the planning of an effect study of this intervention.

The SWG included five patient representatives (three women treated for breast cancer and two men treated for prostate cancer), four researchers and a research assistant. The SWG met four times during the year where the intervention was developed. Data material for the present evaluation study comprises meeting documents, transcriptions of interviews with two patient representatives and three researchers from the SWG, and the primary investigator’s field notes.

The collaboration between patient representatives and researchers informed both the intervention and the research planning and was rewarding for the involved participants. The well-structured organization of the collaboration had a positive impact on the outcome. In addition, clear goals and clarification of expectations were important. Challenges were encountered in keeping continuity between meetings and carrying out homework as intended. It was crucial for the collaboration that patient representatives had specific knowledge, interest and motivation for the project.

Involving patient representatives in the research process heightened the relevancy of the research and the quality of its contents. The SWG gave patient representatives and researchers a better mutual understanding. Overall, the conclusion is that the benefits obtained by involving patient representatives exceeds the additional costs this involves.

**Abstract:**

**Background**

The aim of the paper is to present experiences of researchers collaborating with patients in a shared working group comprising patient representatives and researchers. Experiences are deduced from the evaluation of the work in the working group, which collaborated on developing a psychosocial cancer rehabilitation intervention for women treated for breast cancer and men treated for prostate cancer and the planning of a randomized controlled trial that investigates the effect of this intervention.

**Methods**

Five patient representatives (three women treated for breast cancer and two men treated for prostate cancer), four researchers and a research assistant participated in the shared working group. The shared working group met four times during the year the intervention was developed. Data material for the present evaluation study was collected from meeting documents, transcriptions of interviews with two patient representatives and three researchers from the shared working group, and the primary investigator’s field notes. The data analysis was guided by Sandelowski’s qualitative description strategy.

**Results**

The collaboration between patient representatives and researchers informed the intervention and the research planning and was rewarding for the involved participants. The well-structured organization of the collaboration had a positive impact on the outcome. Also, clear goals and clarification of expectations were important. Challenges were encountered in ensuring continuity between meetings and carrying out homework as intended. It was considered crucial for the collaboration to recruit patient representatives with specific knowledge, interest and motivation for the project. The direct costs related to the shared working group, including meals, transportation and salary for the research assistant, were small. However, the indirect costs in terms of time spent on planning patient-involving elements of, organizing meetings and evaluation were substantial and demanded a significant amount of extra work for the primary investigator.

**Conclusion**

Involving patients in the research process heightened the relevancy of the research and the quality of the research contents. The shared working group influenced both patient representatives and researchers and gave them a better mutual understanding. Overall, the conclusion is that the benefits obtained by involving patients exceed the additional costs related to patient involvement.

## Background

This paper presents experiences and reflections from a shared working group (SWG) of patient representatives and researchers and is reported according to the revised Guidance for Reporting Involvement of Patients and the Public (GRIPP2) checklist [1]. The SWG collaborated on the development and evaluation of internet-delivered Mindfulness-Based Cognitive Therapy (I-MBCT). I-MBCT is an 8-week intervention based on the manual on Mindfulness-Based Cognitive Therapy (MBCT) [2]. In the present project, the I-MBCT intervention was modified to fit the cancer population and aimed at reducing symptoms of depression, anxiety, and stress among women treated for breast cancer and men treated for prostate cancer. The purpose of this paper is to contribute to the exchange of experience with patient and public involvement (PPI) in research and to inspire and guide research peers who wish to involve patient representatives in their own research projects.

The past decade has seen growing interest in involving patients in research and service planning. For example, the English national advisory group, INVOLVE, defines PPI in research as *“research being carried out ‘with’ or ‘by’ members of the public rather than ‘to’, ‘about’ or ‘for’ them”* [3]. This approach contrasts with previous approaches to research and treatment where healthcare professionals considered themselves to be the only true experts for making decisions. PPI contributes with a different perspective than the one held by researchers and clinicians. Hence, PPI may inform the relevancy of the research at all stages, heightens the applicability of information material for study participants and strengthens the quality of implementation [4, 5]. Furthermore, the involved service users report feeling valued and express personal benefits, and researchers report gaining further insight into their research area and a strengthened relation to the community [4, 5]. Challenges in PPI in research are that PPI procedures are time-consuming and costly; moreover, it requires a certain measure of project flexibility to enable change and avoid a tokenistic involvement of patients [5].

Adopting PPI in research requires adaptation of existing methods for developing research projects and interventions or development of new methods [5, 6]. Methods and philosophy of involving patients vary across research fields and traditions [7]. Patients can be involved in all phases of a research process; for example, they can have a say in formulating the research project, adjusting procedures and information material for participants, communicating results to the public, and influencing the implementation of new procedures. In general, patients find it most relevant to be involved in processes closely related to their everyday life as patients and in processes where their influence will matter [8]. Challenges reported by previous studies reporting on PPI in research include patient frustration with lengthy processes when being involved in research; resource demands in terms of time, costs, and additional work; and concerns about irrelevant inputs from patient representatives [7].

Assessment of the effect of PPI in research is scarce and often limited to the intrinsic value for the involved patients and researchers and it rarely details what impact PPI had on the project [9]. Furthermore, the focus of most previously published studies in the field of PPI is on the overall project, not the role of PPI in the research [5, 7], which limits transmission of practical experiences to other studies.

### New methods for developing psycho-social cancer rehabilitation

Cancer survivorship is a field seeing a surge in interest in involving patients to improve planning and execution of intervention and research projects [10]. A meta-analysis of previous studies found that mindfulness-based therapies, such as MBCT, can decrease symptoms of depression, anxiety, and stress among cancer patients and cancer survivors [11]. Although the intervention appears to be successful, its implementation has shown to be hampered by low uptake rates and low adherence [12]. Drop-out rates are reported up to 40%, mainly because of scheduling conflicts, cancer-related treatment or complications, and health-related problems [13, 14]. Internet-delivered therapy is more flexible and widely available than its face-to-face counterpart [15]. In the present project, we developed I-MBCT, expecting the internet format to overcome some of the barriers patients experience in traditional therapy. However, the internet format could create other barriers not foreseen by the professional research group. Therefore, to improve the quality, suitability, and compliance with the I-MBCT program and the feasibility of a randomized controlled trial (RCT) of the program, we invited patient representatives to participate in a SWG with researchers to contribute to the development.

The objective of the SWG was to optimize the I-MBCT intervention and to ensure that information, treatment, and evaluation processes were tailored to the needs and requests of future health-service users from the cancer survivor population. In the present article we focus on the research question: “What did we learn from involving patients in the planning of I-MBCT and the effect study of it?”, and we address the following topics:

- An evaluation of the procedures used for involving patient representatives based on experiences from the SWG.
- Reflections upon the results regarding the impact of the SWG on the project, leading to recommendations for future projects wanting to involve patients in the research process.

This article has a pragmatic approach and intends to describe and inform practice.

The evaluation of the randomized controlled trial that was the subject of the collaboration will be described elsewhere when the study is completed.

## Methods

The evaluation of the PPI work in the research project was performed 15 months after the final meeting in the SWG.

### Participants and setting

Patient representatives were involved in the research as members of a SWG with researchers. The SWG consisted of five patient representatives, two men treated for prostate cancer and three women treated for breast cancer; four researchers, and a research assistant, all women. The primary investigator and the research assistant planned and managed the meetings, whereas the remaining researchers and patient representatives participated equally in the meeting activities, i.e. they got the same meeting invitations and prior information, and participated in the same activities during the meetings, contributing equally with their respective experiences. The breast cancer patient representatives were recruited from previous studies in the research department, including MBCT for persistent pain [13], internet-delivered cognitive training for cognitive impairment [16], and internet-delivered cognitive behavioral therapy for insomnia [17]. The two prostate cancer patient representatives were recruited from the local prostate cancer patient association. Patient representatives were between 49 and 69 years old at project initiation and held educations ranging from vocational training to master degree.

Meetings within the SWG were held in a meeting room at the Department of Psychology, Aarhus University, Denmark, which was also the home of the research project. All participants lived within 20 km from the university. The meetings took place during out of office hours (5 pm to 9 pm) and during each meeting a meal was served. At the first meeting, a briefcase with project documents was handed out to each participant together with a nametag. The SWG was asked to contribute to the formulation of an interview guide, information material, program material, the name of the intervention, to qualify knowledge from interviews, to structure the project procedure, and contribute to the mindfulness program.

### Data

Data was collected from three domains: i) Interviews with members of the SWG were conducted subsequently with five participants from the working group to explore their experience. A researcher who was not part of the original working group (second author) carried out the interviews. Transcriptions from the audio-recorded interviews form the basis of the evaluation of the process and the personal experiences from the SWG. ii) Meeting documents including material sent prior to meetings, meeting agendas, meeting summaries, and e-mail correspondences between patient representatives and researchers. iii) Primary investigator’s reflective field notes about the actual changes made in light of the collaboration in the SWG, based on the original research protocol, draft for information material, draft for program material, and procedure descriptions. The field notes were used to verify the statements in the interviews due to the long time span between end of work and time of evaluation interviews and to explain details in changes in the project that only the primary investigator would have knowledge about.

### Data analysis

The study was analyzed in NVivo 11 guided by Sandelowski’s qualitative description strategy [18–20]. Qualitative description is a suitable analysis strategy for incorporating different data sources in the analysis – in this case interview transcriptions, documents, and reflective notes. Thus, we made a comprehensive summary of the work in the SWG, maintaining the terms used throughout the work. Furthermore, following the naturalistic, inductive inquiry suggested by the qualitative description method [19] we generated six overarching themes from data: *Conflicts of interest*, *A priori considerations about research methods*, *Experience of participation*, *Atmosphere*, *Output*, and *Meeting structure.* The first author conducted the coding, identified codes were then discussed with the second author, and thereafter the structure was presented to the research team for verification. Subsequently we provided a straight description of the phenomena in focus, namely the collaboration between patient representatives and researchers, focusing on process evaluation of the work in the SWG, and the impact the work had on the overall project and on the patient representatives and researchers.

## Results

The results are organized in a structure that reflects the different aspects of organizing the SWG and the experienced impact of the SWG.

### Establishing the SWG

**Fig. 1:**
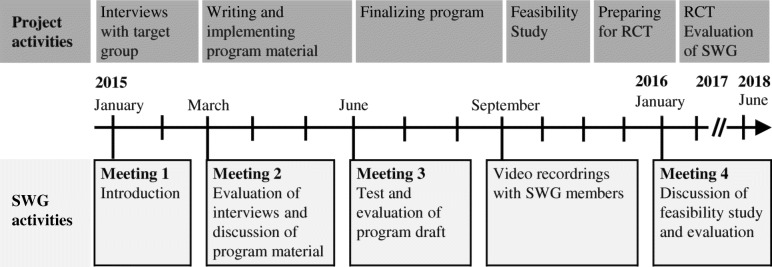
Timeline and overview of project- and SWG activities

An overview of the work in the SWG and how it corresponded to the progress in the overall project is shown in Fig. 1. Activities carried out on the meetings and evaluation of them are described in detail in Table 1.

**Table 1:** Overview of meeting activities in the SWG and their evaluation

Time	Agenda/Activities	Objectives	Evaluation
Material sent out before Meeting 1: - Agenda - Information about project			
Meeting 1 January 2015 Themes: Introduction to the work. Preparation of interviews.	Presentation: About the project	Introduce background of the project.	
	Introduction of group members – “check-in” session.	Introduce all members, establishing sense of equality.	One patient representative mentions in an interview that formal discussions could have been held during dinnertime to shorten the meeting program and that the meetings in general could have been more efficient by cutting down on the more personal parts. Another patient representative mentions in an interview that it was good to be able to talk more informally between the structured meeting activities. Researchers in general comment in the interviews that it strengthened the sense of equality that all participants shared what was going on in their lives at the moment.
	Mindfulness exercise.	Create the right atmosphere for working with a mindfulness intervention and introducing mindfulness for those not already familiar with it.	SWG members (members) appreciated the exercise as exemplification of contents of the intervention. Some liked doing the exercise and others did not find it commendable for them. One researcher mentioned that doing the exercise contributed to the sense of community.
	Framing the work of the working group – “Circle of Control”.	Establish a clear definition of the scope of the project – what can be affected and what cannot. (Fig. 2).	The model worked out very well to clarify the task and expectations and to help members stay focused on the work.
	Match of expectations: “What benefits do you want from the project” and “How can you contribute to the project?”. Personal reflection and plenary discussion.	Ensure a match of expectations before initiating the collaboration and create a sense of ownership in the project.	This activity was not considered very important for the patient representatives in the interviews, but researchers interviewed found it crucial to give opportunity to members of sharing their expectations and also get knowledge about patient representatives’ incentives for participating.
	Dinner and time for informal discussion.	Getting to know each other, heighten the sense of equality among participants, and show appreciation of people spending their free time on the project.	It was agreed by all members that the informal and delightful meal was an important part of the meetings and helped create a good atmosphere.
	Exercise from interview guide: “What term should be used to describe a person in your situation?”. Free discussion and discussion over pre-defined labels, e.g. “cancer survivor”, “women treated for breast cancer”, “previous cancer patient”, “cancer patient”.	Test how the exercise would work.	It became evident that the terminology was important to the patient representatives. There was general agreement that the Danish translation of the word “cancer survivor” was not suitable for how the patient representatives experienced their own situation. This opinion was also dominant during the focus group interviews and individual interviews. The term “woman treated for breast cancer” and “man treated for prostate cancer” were the most favorable terms and were hence used throughout the project for describing the target group for the project.
	Discussion about interview guide.	Have patient representatives’ perspectives on the questions and wording of them.	This discussion qualified the interview guide and allowed the research group to do crucial adjustments. During the discussion, it became clear that patient representatives are not merely patients, but also carry along “dual competencies” consisting of professional qualifications and other experiences.
	Discussion about name of the program.	Find a name for the program matching patient representatives’ preferences.	The researchers thought that it would be important for the patient representatives what to name the program, but they were clear about the fact that they did not find this matter very important. Nevertheless, a suggestion from one patient representative ended up as the popular name of the project.
Material sent out before Meeting 2: - Agenda - Meaning condensation of interviews			
Meeting 2 March 2015: Themes: Evaluation of interviews. Preparation of program.	“Check-in”.	Possibility of sharing state of mind; personal, professional, disease-related or not, of own choice.	(Evaluated above)
	Discussion of results from interviews.	Get patient representatives’ perspectives on the interviews.	Patient representatives made it clear that they did not find themselves capable of analyzing meaning condensations from interviews; they did not feel they had the necessary skills – this was a job for the research group.
	Presentation: Introduction to I-MBCT as a program.	Increase the knowledge about the program in charge in our project.	The presentation was valued by patient representatives and gave an improved understanding of the contents of the intervention.
	Dinner and time for informal discussion.		(Evaluated above)
	Discussion about video material.	Get patient representatives’ views on video material.	Comments on how video material was regarded by patient representatives were very useful for adjusting. Comments focused on the presenter’s appearance in the video. In general, a known professor was preferred over an unknown PhD student to emphasize the authority of the message being delivered.
	Discussion about ethics and security in the program.	Get patient representatives’ views on safety concerns from the user’s perspective.	This matter was of high concern among the researchers, but not of very high interest among patient representatives, who relied on the research group to be in control of things.
Material sent out before Meeting 3: - Agenda. - Information material for participants in RCT. - Login to I-MBCT. - Questions to consider when looking at I-MBCT.			
Meeting 3 June 2015 Themes: Presentation of program draft, discussion of information material.	“Check-in”.	Possibility of sharing state of mind; personal, professional, disease-related or not, of own choice.	(Evaluated above)
	Presentation: Project Status.	Updating members on the status of the project.	
	Revisiting “Circle of Control”.	Ensure a continued focus on the scope of the work.	It appeared to be a simple and straightforward way of ensuring the focus on the task.
	Comments on beta version of I-MBCT.	Initial comments from patient representatives’ perspective.	Patient representatives gave suggestions for how to improve the design of the program by adding visual elements and commented on the length and wording of the texts.
	Comments on information material for study participants.	Ensuring that all relevant information is covered and that it is understandable.	Patient representatives asked questions about wording and structure of the information material and raised crucial understanding issues.
	Dinner and time for informal discussion.		(Evaluated above)
	Planning further development.	Delegation of tasks and agreement about video recordings.	
	Discussion about project procedures; recruitment and information material. Based on personas.	Exploring the way through the study from fictitious patients’ (personas) perspectives.	The outcome of the exercise was useful and gave some very good insights into the process of being a patient. The discussion was based on the patient representatives’ own experiences, not the imagined experience of the personas.
Material sent between meetings: - Test login to I-MBCT. - Feedback template and request for feedback on program.			
Material sent out before Meeting 4: - Agenda			
Meeting 4 January 2016 Themes: Discussion of feasibility study. Evaluation of work in the SWG.	“Check-in”		(Evaluated above)
	Discussion: Comments on the program.	Evaluation of program based on participants testing it at home.	Only few members logged in a couple of times, and no feedback was given in the intended form. The researchers’ own reflections in the interviews are that the task was too unspecified and too comprehensive. One patient representative mentions in an interview that it was probably too much to ask for and that it would have probably worked better if all had met and jointly tested the program. One researcher mentions in an interview that it would have been beneficial to do an extra meeting with the purpose of testing the program together.
	Discussion: Comments on video material for program.	Evaluation of video material.	Overall, the video material received positive response.
	Presentation: Results from feasibility study.	Sharing results from the work we have been doing.	Patient representatives found it interesting to hear about the results from the feasibility study.
	Dinner and time for informal discussion		(Evaluated above)
	Evaluation: Write down “good”, “could be better”, “good advice” on green, red, and yellow post-its. Evaluation on: Level of information, My own cancer illness, Workload, Meetings, Contribution to project, Other.	Evaluation of work in the SWG.	The method for evaluation worked out well as it allowed all members to contribute with what was on their mind. The evaluation of the specific topics is described elsewhere in this paper.
	Presentation of conference abstract about the work in the working group.	Sharing results of research conducted on the basis of the working group in the official form.	One researcher quotes a patient representative’s reaction to the abstract: “Well, it is right that this is what we have been doing, and that is OK, but where is the magic?” (researcher, interviewed). The statement underlines that not only researchers but also patient representatives have experienced a special atmosphere in the collaboration that is beyond the formal work done in the working group.

The PPI in the present project unfolded as a part of the requirements to obtain grant funding the project [10]. The primary investigator experienced that as the planning of the project proceeded, the relevance of patient involvement increased. One researcher interviewed stated concerns she had before initiating the collaboration about involving patients in planning the RCT: “*I thought it could be interesting, but it could also be troublesome. What if they [the patient representatives] came up with a lot of ideas and suggestions that would not be feasible, and it would be much more chaotic. So it was both with an interest in how it would turn out, but also with the concern that it could be much more chaotic than usual when initiating projects*” (researcher, interviewed). Despite a limited availability to possible patient representatives, effort was made to find patient collaborators for the project who seemed to be *“pleasant collaborators, because it is a lot of work together with someone, if they are strenuous …, un-constructive, and too much have their own agenda that moves in an opposite direction… So I’m pleased that we found some good representatives”* (researcher, interviewed). It was also mentioned by a researcher in an interview that the patient representatives from the local patient association were familiar with and interested in volunteer work and therefore had experiences in contributing to initiatives from which patients would benefit.

In the planning of the work, the researchers intended to accommodate the needs of the patient representatives and minimize schedule conflicts for all participants by arranging the meetings during out-of-office hours. Furthermore, few and long meetings were organized to maximize the profit from each meeting and in order not to overburden the working group members with too many meetings. The same structure recurred at each meeting as descried in Table 1. A folder with project materials was distributed to give an organized overview with the possibility of keeping additional project material collected. Uniform nametags were provided for all members to avoid the awkwardness when people do not remember each other’s names. The intention was to facilitate the meetings for a better benefit for the project and to show appreciation and seriousness in the collaboration between patient representatives and researchers by structuring the meetings in a well-organized way.

### Evaluation of meeting structure and process

**Fig. 2:**
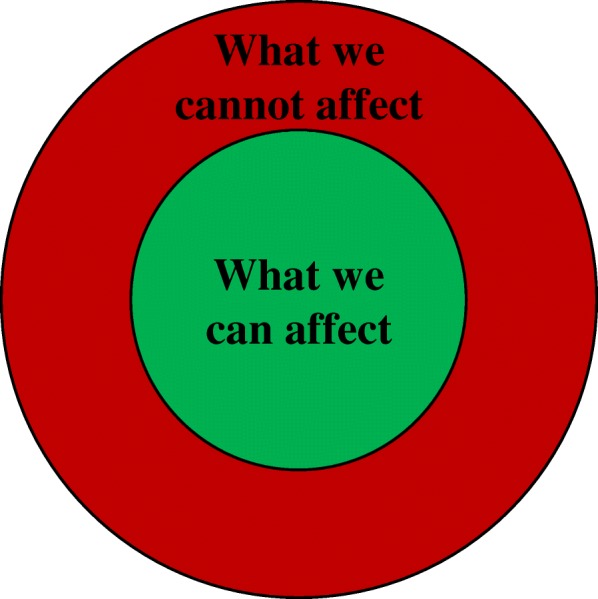
The Circle of Control

Previous studies [7] have identified challenges with collaboration if patient representatives have preferences or wishes that conflict with the project purpose, research methods or simply are beyond the project’s scope. The model “Circle of Control” (Fig. 2) shows which parts of a project can be affected and which cannot. The Circle of Control was introduced and revisited several times during the work to address possible issues with comprehension, to focus the effort to benefit the project the most and to ensure transparency. The Circle of Control was presented to the SWG with notes about different project elements placed in the red or green area to show what influence was possible. The Circle of Control was accordingly used to limit and focus the discussions of the different project elements. As an example, the target population (women treated for breast cancer and men treated for prostate cancer), the overall design of the platform and the manual-based structure of MBCT were placed in the red circle as these were predefined for the project. Opposite these elements, wordings of the treatment content in I-MBCT, recruitment procedures and information material were placed in the green circle as these were subject to the work in the SWG. Control should in relation to the Circle of Control be understood as project elements that was (or was not) subject to actual influence from the SWG’s input and reflections. The final decisions about the different project aspects were made by the responsible researchers complying with the input from the SWG to the best possible extent.

When evaluating the meeting structure, all SWG members commented on the management of the meetings as positive. It was described that the chair of the working group was very friendly and organized the meetings in a pleasant way: “*She stayed focused without being dominating*” (researcher, interviewed) and *“I think she was good at explaining what it was all about and she was very empathic throughout the project. Kind to listen and ask. She was maybe a bit wavering in the beginning, but it was maybe also new for her to do something like this (PPI collaboration)”* (patient representative, interviewed*).* The role of the research assistant was also appreciated as an important role in the facilitation: Commenting on “something that was in particular good”: *“[the research assistant] took minutes. She kept the overview and held on to what we were discussing… she was good at it.”* (patient representative, interviewed)*.* The framing with meeting structure, briefcases and dinner was acclaimed by the researchers as a significant planning element: “*…the planning and management of the meetings meant a lot for the outcome*” (researcher, interviewed) and “*...what I primarily have learned from this is something about framing… it doesn’t only apply for user involvement in the development of something, it applies in general when you meet patients*”(researcher, interviewed). During the discussions, it became clear that the patient representatives were very willing to share their points of view upon the different topics, and that the tasks really mattered to them. A researcher commented in an interview on the interview exercise ‘What should we call people like you?’ (Meeting 1): “*And that is where it became meaningful. When you discuss something that is important to people and they actually dare to tell what it means to them… That it is essential topics that are brought up*” (researcher, interviewed).

Patient representatives argued in the interviews that *“I think too long time was passing between the meetings…[I think] a maximum of three to four months between the meetings, otherwise I almost can’t remember it… and the problem is then that it becomes unimportant because one is not retained”* (patient representative, interviewed). The researchers, on the other hand, were working on the project on a daily basis and did not need to refresh their memory between meetings. It was in general agreed that the meetings were too long given the time of the day. SWG members disagreed on whether more or the same number of meetings would be preferred. It was suggested to arrange that some of the SWG members met in between the meetings to conduct specific activities (e.g. making small videos for the program). Assignments such as activities to be carried out at home were not successful. One researcher elaborated in an interview on having the SWG members testing the program at home: “*You cannot expect that people will do a lot of work between the meetings. It is like the students. They do not work so much between the meetings, so those things where you want input, should be done during the meetings*” (researcher, interviewed). A patient representative had similar reflections and corresponding suggestions: *“I simply think that we did not have the time. They could have told us to bring our laptops [to a meeting] and connect us there. And then we could go through it [together]”* (patient representative, interviewed). It was mentioned that earlier involvement of patient representatives could possibly have had a stronger, positive impact on the project. In the present project, many decisions were already made before involving the patient representatives, which limited the room for improvement. The Circle of Control (Fig. 2) was, however, an effective method to specify the scope of the collaboration.

It was the researchers’ intention that the members of the SWG should have a pleasant experience as collaborators in the research project and feel encouraged to contribute with whatever was on their mind, but still within the scope of the project. In the evaluation of their experience from participation, all SWG members agreed that a sense of equality characterized the work. The check-in session in the beginning of each meeting was described as contributing to the sense of equality “*I think it was a good way of doing it. … people were equally important because they were allowed to present what was in progress for them at the current moment also if that actually was not related to the project*” (researcher, interviewed). Equality in discussions was also described: “*I experienced a very big respect for each other’s fields of expertise… there was equality in discussions and relational respect, but no real disagreements on who were experts in this and that*” (researcher, interviewed), a colleague agreed: “*…there was a common spirit. And it was strengthened by the framing, I think. We were all curious to get wiser, and we wanted to spend time on this, to invest in it*” (researcher, interviewed). Researchers were eager to contribute to the work based on their experiences as patients and their professional and personal expertise. Furthermore, they emphasized that when the SWG engaged in activities, they found themselves incapable of, e.g., commenting on interview material (Meeting 2) and explicitly assigned the task to the researchers. One researcher mentioned that due to the presence of both men and women, a special dynamic occurred and that it was important not to isolate one particular patient group (e.g. based on gender or disease) within the group. “*Sometimes it was just a different way to talk about it than the way women talk about it… A different way of using the words*” (researcher, interviewed). Another issue raised during the interviews was that it was challenging to recruit men for the ongoing study. The male patient representative underlined the importance of offering interventions to men despite their low attendance rate: “*So here it is revealed [that men think that] “I don’t need this”. But that is rubbish… Because men have just as many problems as anyone else*” (male patient representative, interviewed).

The direct costs of involving the SWG in the planning of the project are described in Table 2. In addition to the direct costs, the primary investigator undertook a considerable amount of work to restructure the research project to encompass the PPI structure. Hence was one comment answering the question “what did you get out of involving patients in the research project”: “*…it gave me some extra work*” (researcher, interviewed); however, it was also stated by the researchers in the interviews that despite the extra amount of work, it was still worth involving the SWG.

**Table 2:** Direct costs of PPI in the research project

Item	Cost description
Research assistant	
Meeting preparation	3 h × 4 meetings
Meeting participation	4 h × 4 meetings
Writing meeting summary	2 h × 4 meetings
Participants (5 patient representatives and 4 researchers)	
Meeting preparation^a^	1 h × 4 meetings
Meeting participation^a^	4 h × 4 meetings
Venue	
Meeting room at Aarhus University, Denmark^b^	With av-equipment and suitable arrangement
Catering	
Dinner	10 persons × 4 meetings
Coffee and cake	10 persons × 4 meetings
Stationery	
Name tags	10 pcs
Folders	10 pcs
Copies of slides	10 copies × 4 meetings
Other	Post-its, pens, notepads

^a^Patient representatives were offered compensation for transportation costs, but none of them accepted the offer because all lived within 20 km from the venue. Furthermore, patient representatives were not payed for their participation in the SWG as well as researchers were not offered overtime payment

^b^The meeting room was available without additional cost for the project

### Evaluation of project impact and personal output from the SWG

The key changes, based on input from the SWG, relevant to the I-MBCT program and the research project are described in Table 3. Overall, the changes in both the I-MBCT program and the research project were related to a user-friendly wording of text and examples and procedures adjusted for the specific population of women treated for breast cancer and men treated for prostate cancer.

**Table 3:** Changes applied to the I-MBCT program and the research project based on the collaboration with the SWG

Key changes in I-MBCT program	Examples
Wording of text has been adjusted to fit the patients’ preferences and for better understanding.	- The text was shortened and organized in smaller sections to increase clarity. - Psychological terms (e.g. “cognitive”) were explained.
Examples mentioned in the program have been added and tailored to fit the preferences of the patient representatives.	- Examples were made cancer specific targeting recognizable experiences related to cancer treatment and late-effects instead of being generally related to psychological distress.
The visual structure of the program was modified. Colors and graphical explanations were added to facilitate understanding and focus.	- Instead of having the program material in running text, the content was structured in information boxes, exercise boxes and example boxes with different identifiable layouts, e.g. blue for information, yellow for exercises and speech bubble for participant examples.
Video examples were made with patient representatives from the SWG to supplement videos with expert statements. Video organization was based on patient representatives’ preferences.	- Three patient representatives from the SWG participated in the videos talking about their own experiences with living with cancer late effects and previous participating in Mindfulness-Based Cognitive Therapy.
Key changes made to the research project	
Specific adjustments were made to the interview guide for the initial interviews with women treated for breast cancer and men treated for prostate cancer. Questions were re-worded for a better understanding	- Clarification of the purpose with the interviews was emphasized. - Language was modified to be less legalese. - Examples of some of the themes (e.g. how cancer survivors should be addressed in Danish and existing online discussion forums) was printed on paper to show in the interviews.
The recruitment procedure, targeting study participants from the hospital outpatient clinic, was justified.	- Other possible recruitment procedures were discussed (e.g. online advertisement on social media and leaflet at the Oncology Department), but the original planned procedure was maintained because it was stated as preferable as it exudes seriousness to the treatment.
Information material was reformulated and restructured after patient representatives asked questions about concepts they did not understand.	- A clarification of what “Mindfulness-Based Cognitive Therapy” means was added (the term “cognitive” was not understood). - The structure was changed into a different order presenting how the program could benefit the patients first and then research technical details later.
Recruitment procedures in the hospital clinic were revised. The revision was based on the new perspective the patient representatives gave on when and how to invite patients to participate in the study, and how to go about the procedures in the hospital, bearing the patients’ perspectives in mind.	- It became clear that the optimal time for providing a psychosocial intervention was after the primary treatment (chemotherapy, radiation therapy and surgery) was completed and when patients are released from the course of treatment and can feel “left alone with their thoughts”.
Based on comments from the men treated for prostate cancer, it was clarified that sexual dysfunction is an underestimated issue that they experience is not raised among health professionals.	- An additional question about sexual dysfunction was added to the questionnaire regarding late effects.

The researchers had no preliminary intentions that SWG members should gain personal benefit from participating in the working group, but it became evident during the evaluation session and the interviews that it did have a great impact on both patient representatives and researchers. Both patient representatives and researchers expressed in the interviews that it had been meaningful and rewarding to participate in the work. A patient representative described: “*… I think that an educated society like ours should get going more with that, to be frank… I think that it is possible to get something prudent out of something like this…because we provide body and tears and brains and disaster and destroyed lives and pain. Everybody who has gone through it [cancer disease and treatment], too bad*” (patient representative, interviewed). A researcher elaborated on the benefit of PPI as opposed to meeting patients in the treatment context like this: “*It is important to ask the patients in a different forum than the therapeutic one, because there is a huge difference between sitting in the therapy group and going here. It is a different place, and I think that was a smart idea. I think it teaches you something that you cannot really learn in any other way… [it can] optimize…[and] make it more efficient and satisfactory for all parties*” (researcher, interviewed). It was further mentioned that the way the patient representatives talk about and address different issues contributed to other research projects as well. “*Sometimes it can be hard to specifically say what the SWG has changed, but I think it has shaped my way of thinking about research. And every time we have a project where something is not working, I’m thinking, “Why didn’t anyone ask the patients what they prefer?”…I remember that after the first meeting my supervisor...said “…this will revolutionize the way we do research*”” (researcher, interviewed).

Patient representatives expressed that they felt grateful for having the opportunity to influence the healthcare system, which they did not feel was a matter of course in general. Both patient representatives mentioned in the interviews that they would have liked to get more information on the status of the project along the way; not just about the results, but also about the progress.

## Discussion

The aims of this paper were to contribute to the exchange of experiences with PPI in research by evaluating the procedures used for involving patients in the SWG and their reflections on the impact of the SWG. Overall, the PPI in the present project was considered successful, and deliberations on the reasons for this are presented in the results section above. Our main findings were: i) The organization of the collaboration had a strong impact on the outcome. ii) It is important that researchers clarify the purpose of involving patients as collaborators and present this purpose in a clear manner, for example by use of a “circle of control”. iii) The workload associated with PPI in the research project significantly exceeded the direct costs. iv) The effect of the working group related to both changes in the project and personal experiences of members of the SWG. Furthermore, a central point that will be further discussed below was that identifying and recruiting patient representatives with specific knowledge, interest, and motivation for the project was crucial for the collaboration.

### Establishing the SWG

Finding and selecting appropriate patient representatives that are able and willing to engage in collaboration on the project’s premises was considered important by the RIs. As the I-MBCT project builds on previous projects for women treated for breast cancer from our research department, patient representatives were recruited among previous participants from these projects and from a local patient association to also involving men treated for prostate cancer. The difficulty of recruiting relevant patient representatives for the present project is also rooted in ethical issues pertaining to access to relevant patients because others than the assigned doctors are denied access to the medical records; moreover, previous research participants can be contacted only as a part of the study, not afterwards. As is often the case in research, it was challenging to recruit participants from low-income and low socio-economic status groups. This is a possible limitation since we cannot know whether input from less privileged patients would have identified other weaknesses in our materials and design. Although the patient representatives were quite well-reflected on representing themselves as patients, but also representing other patients who were known to them, they might not have been aware of problems encountered by less educated, more distressed, or physically or mentally disabled patients. We noticed that men treated for prostate cancer sometimes offered suggestions and input that were unexpected in the female research group. This suggests that recruiting patient representatives who differ substantially from the researchers is important because they may potentially contribute with the most valuable insights; insights that the researchers cannot foresee when planning the study. An ongoing challenge within the field of cancer rehabilitation (and in other research areas as well) is to recruit male participants [21]. Even though this issue is also present in the evaluation study of the present project, the statements from the men treated for prostate cancer in the SWG underline the importance of offering the intervention and possibly other interventions in the future to men, even though only a few will sign up for them. A possible way of addressing this issue, i.e. before recruitment of male participants becomes a problem, could be to involve men in earlier stages of the project planning.

An ongoing discussion within the field of PPI in research is the importance of educating patients to be involved in research as collaborators. Some (e.g. Sacristán et al., 2016) find that patients should be educated into “expert patients” in order to be able to contribute to a research project at all. Our approach to this has been a very pragmatic one as we initiated the work in the SWG shortly after the patient representatives were recruited; and it would hence have been impossible to have them participate in an education on beforehand. Some patient representatives mentioned that it was difficult to find the time for tasks between the meetings. Our impression is therefore that it would have been even more challenging to find additional time for participating in research education. Furthermore, we consider it as a strength in the present project that we had patient representatives who had gone through similar interventions before and therefore could contribute based on very relevant, recent experience. Our opinion is therefore that as treatment protocols change constantly, it will be challenging to get patients with updated and relevant experiences if they are to undergo education before being able to engage in PPI. If needed for a specific project, patient education can be a part of the collaboration ensuring that relevant research knowledge is taught and to avoid that patients spend additional time. As revealed in the interviews, the framing of the collaborative work was essential for the outcome. The way activities in the collaboration are facilitated may compensate for patient education by including the needed information about research procedures and by formulating activities in a way that patient representatives can understand without a prior research education. Furthermore, we consider it as a strength and a commonplace of the collaboration that researchers are forced to reframe their requests to fit the patient representatives’ prerequisites, not the other way around. Demanding that patient representatives undergo research education before being able to participate in research may have another unwanted consequence, namely that only highly educated patients are able to complete the education and hence participate in the research project. Engaging in the task of reframing the research project and communicating with lay people may furthermore strengthen the researchers’ relation to the surrounding community.

### Organizing the collaboration between patient representatives and researchers

According to the literature, patients can be involved at all stages of a research project [7]. The present study found that it will be preferable for future projects to initiate the collaboration earlier than we did in the present project, because many things were decided on beforehand (the intervention, the target group, and the digital platform), so relatively few things were possible to change. It might have been preferable to involve patients beforehand, i.e. in the research design and project planning phase to ensure that patients’ wishes permeate both intervention, target group, delivery method (i.e. face-to-face, group or online), and study design. However, as seen elsewhere [22], it can be a project in itself to involve patients in prioritizing subjects for further research. On the other hand, when inviting external collaborators for a research project, part of the scope should be decided in advance to ensure that patient representatives would have specific tasks to respond to. Furthermore, it will probably be very challenging to raise funding for a project if the research design is not decided upon beforehand. During the work, some patient representatives contributed to the discussions based on their professional background (e.g. within communication), which was not distinctly expected of them; yet, it was, indeed, helpful for the project. Dealing with these “dual competences” can be beneficial for the project, and patients should be welcomed as complex individuals contributing based on their multiple experiences, not only as a person with a cancer label. However, attention towards how the dual competences are utilized should be drawn to prevent exploitation.

The literature also stresses the importance of avoiding that PPI becomes tokenistic and are initiated only to accommodate external demands [5]. We made a great effort to maximize the level of transparency in the work to ensure that patient representatives knew what the SWG could influence. Furthermore, we prioritized involving the patient representatives in tasks and at stages where there was actual room for improvements. In a strict research design, it takes some effort to unfold possible project aspects where patients’ input can be beneficial. Our experience was, however, that we could ask patient representatives literally anything that involved them during the project process, and that they were clear about expressing if they did not think they had anything useful to add. Hereafter it was the researchers’ responsibility to adjust the project to accommodate the patient representatives’ requests but also to fulfill research methodological requirements. Our impression is that conflicts of interest between researchers and patient representatives that occurred during the discussions sometimes can be handled by using clear and factual reasoning on how research methodological demands can be met without compromising patients’ statements. In the present collaboration, we did not experience disagreements that could not be solved in consensus. Given that the health care system, including research projects, deals with very important aspects of patients’ lives, situations may occur where emotional beliefs towards aspects in the research project from both patient representatives and researchers that cannot be solved in consensus. In these situations, we believe an acknowledgement of the disagreement is necessary and that this disagreement should be taken into account in the subsequent work. A part of the success of the SWG was attributed to meeting facilitation, encompassing clarity on the tasks and transparency. Both researchers and patient representatives expressed in the interviews, that the collaboration between researchers and patient representatives added a different but important dimension to the researchers’ understanding of patients’ world. The understanding differs from the understanding achieved during regular clinical contact. This particular meeting and knowledge exchange between patients and researchers can be understood as the participatory space described by Renedo & Marston [23]. Creating a suitable participatory space is crucial for the success of PPI [23].

We think that using “the circle of control” as a recurrent tool has helped focus the tasks at meetings and created a transparent framework for the SWG’s overall purpose. In future PPI projects, we recommend introducing a “circle of control”, but the research group needs to consider carefully which specific conditions are subject to change and which are not.

From the time when the work in the SWG was initiated until now, much has been thought, written, and developed within the field of PPI in research in terms of guidelines, attitudes and theories [24, 25]. Especially attention can be drawn to the health care system in the UK that is in general experienced with PPI in research [26, 27]. The recently launched standards for PPI from the National Institute of Health Research [27] can be inspirational for others, bearing in mind that differences may occur across different health care systems internationally and across contexts [25].

### Costs

Even though the direct costs of the work in the SWG are not huge, our experience is that it is resource demanding to involve patients in a research project. This finding corroborates others’ findings [5, 7]. PPI in research requires time and resources for establishing the collaboration, preparing and conducting meetings, and for adjusting the project. Before initiating PPI, the research group and the project stakeholders should agree that it is a priority to involve the patients in collaboration and should be ready to bear costs both economically and time-wise. From our point of view, and as also stated by Pizzo et al. [28] in their elaboration of the costs and benefits of PPI, spending resources on PPI can be seen as an investment in raising the level and relevancy of the research being conducted.

### Impact of the SWG

Most of the requests from the research group were fulfilled. The intention of getting patient representatives’ perspective on the results from the interviews with women treated for breast cancer and men treated for prostate cancer and a more thorough feedback on the I-MBCT program should, however, have been facilitated in a different way to increase output. Our own suggestion would be to simply make sure that all important work was carried out during the meetings and not between meetings, to ensure clarity about the task, and avoid time constraints for the SWG. Furthermore, shorter meetings (considering the evening format), shorter intervals between meetings, and regular updates on the project would have been preferred to ensure maximum impact of the SWG. We think that the most interesting finding from the work was how much impact the SWG had on the researchers’ way of thinking about research projects. Not only did the working group contribute to the specific tasks they were assigned, but the dialogue between patients and researchers raised the way of thinking about the research projects to a new level. This realization was not expected by the researchers on beforehand, but has recently also been described elsewhere [24]. We believe that the impact of PPI in research on researchers’ attitudes towards patients and research in health care benefits beyond the specific impact of PPI and is of crucial value to the health care system.

### Limitations

The material that the present study is based on is influenced by the fact that the initial intention of the SWG was to inform the overall project, not to evaluate the work itself. For future projects involving patient representatives in the research, it would be strongly recommendable to incorporate the evaluation of the collaboration between patients and researchers from the outset. The work of Staley et al. [25] concerning “realistic evaluation” dealing with the interrelated aspects of context, mechanisms and impact sets a very usable frame for this work. Planning the assessment and dissemination of the PPI in research is furthermore in alignment with the recently published GRIPP2-guidelines [1].

Due to the post hoc decision to evaluate the work in the SWG the interviews with the members was not performed until one year after the last meeting in the SWG, which may have introduced recall bias. Some of the researchers had reflected upon the work in the SWG and had already implemented some of the procedures in new research projects to give patients’ voice a larger say. These reflections and experiences would not have been present if the evaluation had taken place immediately after the conclusion of the collaboration, which supports the relevancy of the evaluation study, despite the time delay.

## Conclusion

Based on our experiences from the present project, our main conclusion is that involving patients in the research process can contribute to heightening the relevancy of the research in the community and the quality of the research contents for the research participants. Furthermore, it can strengthen researchers’ and patient representatives’ mutual understanding. Our findings underline the importance of researchers clarifying the purpose of involving patients as collaborators and to ensure that the purpose is presented for the patient representatives in a clear manner, for example by use of a “circle of control”. Furthermore, our experiences suggest that selecting suitable patient representatives for the purpose of the collaboration is of crucial importance to ensure a productive and constructive partnership. The length of meetings and intervals between meetings should be shorter than in the present study to accommodate the participants’ needs, avoiding exhaustion during meetings, and strengthening project cohesion. Furthermore, important work should be placed within meetings and work at home be kept to a minimum. In the facilitation of the meetings, our impression is that an effort to create a sense of equality among members helped the discussions flow more freely and encouraged patient representatives to contribute based on their true requests. When planning PPI in research, we strongly recommend planning the assessment and dissemination of the impact of PPI from the beginning of the project, as sharing experiences among researchers within this emerging field is very valuable. In budgeting for PPI in research, awareness of the extra workload related to PPI should be considered in addition to the direct costs of the meetings. From our point of view, the importance of engaging the patients who have “*…provide[d] body and tears and brains and disaster and destroyed lives and pain. Everybody who has gone through it [cancer disease and treatment].*”(patient representative, interviewed) far exceeds the additional workload and costs associated with PPI.

## Abbreviations

I-MBCT: Internet-delivered Mindfulness-Based Cognitive Therapy
MBCT: Mindfulness-Based Cognitive Therapy
PPI: Patient and Public Involvement
RCT: Randomized controlled trial
SWG: Shared Working Group
